# Patterning two-dimensional free-standing surfaces with mesoporous conducting polymers

**DOI:** 10.1038/ncomms9817

**Published:** 2015-11-18

**Authors:** Shaohua Liu, Pavlo Gordiichuk, Zhong-Shuai Wu, Zhaoyang Liu, Wei Wei, Manfred Wagner, Nasser Mohamed-Noriega, Dongqing Wu, Yiyong Mai, Andreas Herrmann, Klaus Müllen, Xinliang Feng

**Affiliations:** 1 School of Chemistry and Chemical Engineering, Shanghai Jiao Tong University, 800 Dongchuan Road, 200240 Shanghai, China; 2 Department of Chemistry and Food Chemistry & Center for Advancing Electronics Dresden (cfaed), Technische Universität Dresden, 01062 Dresden, Germany; 3 Department of Polymer Chemistry, Zernike Institute for Advanced Materials, University of Groningen, Nijenborgh 4, Groningen 9747, The Netherlands; 4 Max-Planck-Institut für Polymerforschung, Ackermannweg 10, 55128 Mainz, Germany

## Abstract

The ability to pattern functional moieties with well-defined architectures is highly important in material science, nanotechnology and bioengineering. Although two-dimensional surfaces can serve as attractive platforms, direct patterning them in solution with regular arrays remains a major challenge. Here we develop a versatile route to pattern two-dimensional free-standing surfaces in a controlled manner assisted by monomicelle close-packing assembly of block copolymers, which is unambiguously revealed by direct visual observation. This strategy allows for bottom-up patterning of polypyrrole and polyaniline with adjustable mesopores on various functional free-standing surfaces, including two-dimensional graphene, molybdenum sulfide, titania nanosheets and even on one-dimensional carbon nanotubes. As exemplified by graphene oxide-based mesoporous polypyrrole nanosheets, the unique sandwich structure with adjustable pore sizes (5–20 nm) and thickness (35–45 nm) as well as enlarged specific surface area (85 m^2^ g^−1^) provides excellent specific capacitance and rate performance for supercapacitors. Therefore, this approach will shed light on developing solution-based soft patterning of given interfaces towards bespoke functions.

## Abstract (web summary)

Solution-state patterning of functional materials on surfaces is important for a number of emerging technologies. Here, the authors demonstrate a bottom-up method of endowing freestanding surfaces with mesoporous conducting polymer coatings for enhanced electrochemical capacitance properties.

## Graphical abstract

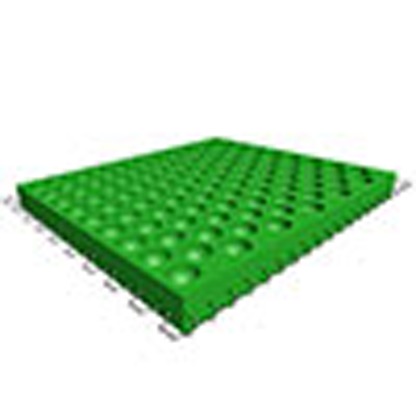

Recent research has shown that the dimensionality of materials plays a crucial role in their fundamental properties. This phenomenon has been highlighted by the 2004 discovery of graphene with a two-dimensional (2D) structure that exhibits exotic phenomena absent in bulk graphite^1^,^2^. Inspired by the striking success of graphene, graphene-like 2D nanosheets and their composites have been widely studied^3^,^4^,^5^,^6^,^7^,^8^. Among them, oxide nanosheets including titania are exceptionally rich in both structural diversity and electronic properties and therefore show great potential in electronics, catalysis, energy storage and conversion^9^,^10^,^11^,^12^. Ultrathin metal chalcogenides (for example, MoS_2_), with a defined bandgap, large on/off ratio and near-theoretical subthreshold swing values, have been widely studied in nanoelectronics. Indeed, these ultrathin 2D free-standing nanosheets with high flexibility and large specific surface areas are exquisite building blocks for constructing hierarchical architectures. Both surfaces of these 2D free-standing nanosheets can be utilized for the growth of functional composites to form unique sandwich structures. Their open architectures and the strong coupling effects within 2D sandwich structures provide materials with remarkable properties for catalysis, solar cells and electrochemical energy storage^12^,^13^,^14^. On the other hand, compared with those traditional patterning techniques on the stationary substrates via lithography or thermal evaporation under harsh conditions, developing new micro/nanofabricating methods based on solution-based soft patterning is more promising for large-scale applications because of their exceptional advantages, such as cost effective, high throughput, mild condition and so on. Despite of being highly desirable, the direct patterning of functional moieties with regular arrays on 2D free-standing ultrathin nanosheets remains a great challenge.

Conducting polymers with conjugated backbones may exhibit good electrical conductivity, high electron affinity and low-energy optical transitions^15^,^16^,^17^. In particular, polypyrrole (PPy) and polyaniline (PANi) have been extensively utilized in energy storage systems, light-emitting diodes and biomedical devices due to their ease of synthesis and processability^15^,^16^,^17^. Recently, different architectures have been designed to improve their performance. Well-defined mesoporous structures can provide polymer surfaces with an interconnected pore network, narrow pore size distributions (PSDs) and large specific surface areas. The combination of a mesoporous structure with the inherent characteristics of these conducting polymers would greatly enhance their performance in electronic or electrochemical devices^15^,^16^,^17^. Although soft-template methods, such as evaporation-induced self-assembly of surfactants, have been well established for the preparation of mesoporous metals, metal oxides and carbon^18^,^19^,^20^,^21^,^22^,^23^, one-step synthesis of well-defined mesoporous conducting polymers by a solution-based self-assembly route has not been achieved. This even holds true 20 years after researchers at Mobil discovered the first mesoporous silica, which is likely due to a lack of suitable structure-directing agents to guide the assembly of monomers as well as the high volatility of monomers^24^,^25^,^26^,^27^,^28^,^29^,^30^.

In this study, we design a universal strategy for solution-based patterning of mesoporous conducting polymers with controlled pore sizes that is assisted by monomicelle close-packing assembly of block copolymers (BCPs) on free-standing 2D surfaces. Amphiphilic polystyrene-*b*-poly(ethylene oxide) (PS-*b*-PEO) diblock copolymers with different PS block lengths and PS:PEO ratios were chosen as structure-directing macromolecules that can self-assemble into spherical micelles in a tetrahydrofuran (THF) and H_2_O solvent mixture. The spherical PS-*b*-PEO micelles closely organize on both 2D surfaces via H-bonding and guide the oriented polymerization of pyrrole or aniline monomers. Upon removal of BCP templates, patterned PPy and PANi with regular mesoporous features on free-standing 2D surfaces were achieved. Taking single-layer graphene oxide (GO) as an example, the obtained GO-based PPy nanosheets possess unique sandwich structures with adjustable pore sizes (5–20 nm) and thickness (35–45 nm), depending on the selected BCPs. This approach can be further expanded for fabrication of mesoporous large-pore conducting polymers on other functional surfaces, including 2D electrochemically exfoliated graphene (EG), MoS_2_ and titania nanosheets as well as on one-dimensional (1D) carbon nanotubes (CNTs). These ultrathin sandwich structures with open and regular pores have unique properties for energy storage, as exemplified by supercapacitor electrodes. The mesoporous PPy@GO nanosheets with the pore size of ∼6 nm and specific surface area of 85 m^2^ g^−1^ show excellent electrochemical performance with capacitance of 383 F g^−1^ at 1 mV s^−1^ and cycling stability with ∼99% capacitance retention after 5,000 charge/discharge cycles, as well as remarkable rate performance for the on-chip all solid-state micro-supercapacitors (MSCs). Therefore, our strategy will pave the way to controlled bottom-up construction of mesoporous 2D hybrid materials for various technological applications.

## Results

### Patterning of 2D mesoporous conducting polymers

**Figure 1:**
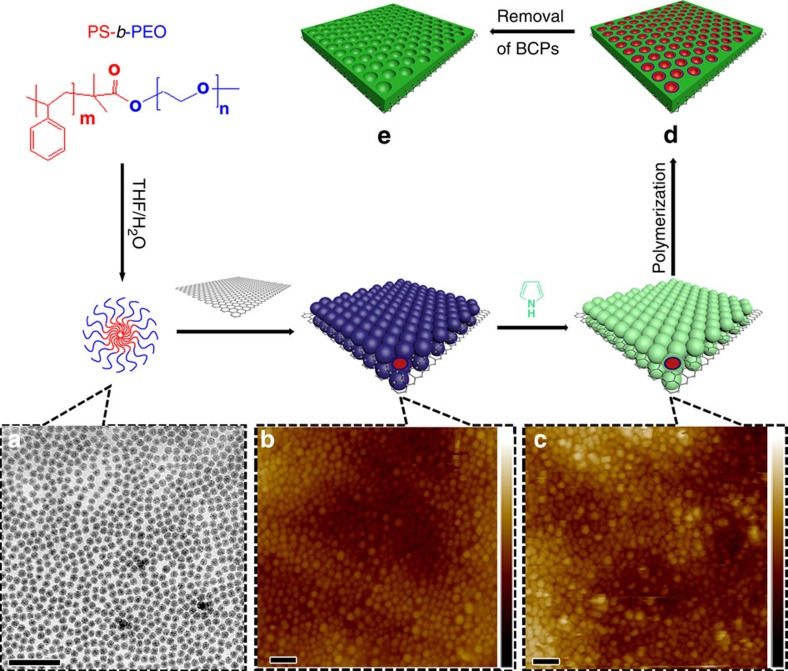
Patterning of 2D surfaces with mesoporous conducting polymer nanosheets. (**a**) Formation of spherical BCP micelles in THF and H_2_O solvent mixture at room temperature and the corresponding TEM image. (**b**) Direct observation of self-assembled BCP micelles on the GO surface by tapping mode of AFM in solution. Vertical scale is 120 nm. (**c**) Co-assembly of BCP micelles and pyrrole monomers on the GO surface and corresponding AFM image. Vertical scale is 107 nm. (**d**) Polymerization of pyrrole monomers on addition of ammonium persulfate initiator. (**e**) As-made mesoporous PPy nanosheets on graphene surfaces obtained after removal of BCP templates (scale bar, 100 nm).

A BCP-directed self-assembly route is proposed to achieve bottom-up patterning of 2D conducting polymer nanosheets with controlled mesopores (Fig. 1). The free-standing single-layer GO sheet in aqueous solution is chosen as a typical 2D surface, given that the abundant oxygen-containing functional groups would guarantee its hydrophilicity and dispersibility in aqueous or polar solvents, thereby facilitating the self-assembly of other functional molecules or nanoparticles onto surface. Amphiphilic BCPs of PS-*b*-PEO with different lengths of hydrophobic PS blocks are selected as directing templates. The pore size of mesoporous materials derived from BCP templates could be easily adjusted to above 10 nm by simply varying the molecular weight of the PS block. Low-molecular-weight surfactants and pluronic-type structure-directing agents typically give rise to pore sizes below 10 nm due to the length limitation of the hydrophobic segments^18^,^23^,^31^. The BCP templates were synthesized via atom transfer radical polymerization^32^. By varying the polymerization time, a series of PS-*b*-PEOs with different PS block lengths were prepared. Their compositions can be described as PS_38_-*b*-PEO_114_, PS_102_-*b*-PEO_114_ and PS_146_-*b*-PEO_114_, according to gel permeation chromatography analysis. Supplementary Fig. 1 reveals that the polydispersity index of these BCPs is below 1.10, indicating a narrow molecular-weight distribution, which would be favourable for the synthesis of mesoporous materials with the uniform pore size and regular pore arrays. The corresponding micelle sizes of PS-*b*-PEO in mixed solvents of THF and H_2_O vary depending on the PS block lengths. Dynamic light scattering characterization shown in Supplementary Fig. 2 indicates that micelles of PS_38_-*b*-PEO_114_, PS_102_-*b*-PEO_114_ and PS_146_-*b*-PEO_114_ have narrow particle size distributions with hydrodynamic diameters of ∼21, ∼35 and ∼54 nm, respectively.

The fabrication of mesoporous conducting polymers of PPy on the GO surface is schematically illustrated in Fig. 1. First, spherical micelles are formed by self-assembly of the PS-*b*-PEO amphiphilic diblock copolymers in a THF/H_2_O solvent mixture at room temperature (Fig. 1a)^33^. Transmission electron microscopy (TEM) survey presented in Fig. 1a verifies the formation of monodisperse spherical micelles with the uniform size of ∼15 nm. This size is smaller than that measured by dynamic light scattering because of the more shallow contrast of PEO shells for TEM investigation. Second, when adding the GO dispersion, the preformed spherical micelles self-assemble and anchor on the GO surface. The direct virtual observation by atomic force microscopy (AFM) clearly illustrates this self-assembly process in solution, as shown in Fig. 1b. Spherical micelles with a diameter of ∼20 nm arrange into single-layered architecture with close packing on the GO surface. In contrast, a close-packing arrangement of PS-*b*-PEO micelles cannot be formed on surfaces of high-quality graphene from electrochemical exfoliation under the same conditions^34^. Therefore, the driving force for assembly is proposed to be hydrogen bonding on chains with the oxygen-bearing groups on GO (such as –COOH and –OH)^35^. This can be confirmed by the sharp increase of zeta potential when adding neutral BCP micelle solution into the GO dispersion (Supplementary Fig. 3), which suggests that the strong H-bonding interaction can lead to the effective adsorption and coating of BCP micelles on the GO surfaces. Third, after pyrrole monomers are added, solution-based AFM analysis demonstrates that single-layer spherical BCP micelles remain in a close-packing arrangement architecture on the GO surface with slightly larger feature sizes (Fig. 1c). Interestingly, it has been observed that the BCP micelles without pyrrole were mobile on GO surface and could be easily dislocated by the AFM tip during imaging. In contrast, in the presence of pyrrole monomers, BCP micelles showed stronger adhesion to GO and did not show partial disassembly during AFM scanning. This result demonstrates that pyrrole monomers around BCP micelles can serve as a linker to further stabilize BCP micelles and ensure their lateral close-packing arrangement into single layer on both GO surfaces. Fourth, after polymerization at room temperature by addition of ammonium persulfate, pyrrole monomers are transformed into polymer networks around the BCP templates^36^. Similar to the Ostwald ripening process^37^,^38^, a strong force from polymerization might introduce the migration of pyrrole monomers along with the PEO chains from the top of spherical micelles into the voids of adjacent micelles^38^, thereby forming mesoporous channels (Fig. 1d). Finally, after removal of BCP micelles by repeated washing with THF, GO-based mesoporous PPy nanosheets were obtained (referred to as mPPy@GO; the mesoporous GO-based PPy nanosheets synthesized by PS-*b*-PEO with different PS block units of 38, 102 and 146 were denoted as mPPy@GO-1, mPPy@GO-2 and mPPy@GO-3, respectively).

### Structure of GO-based mesoporous conducting polymers

**Figure 2:**
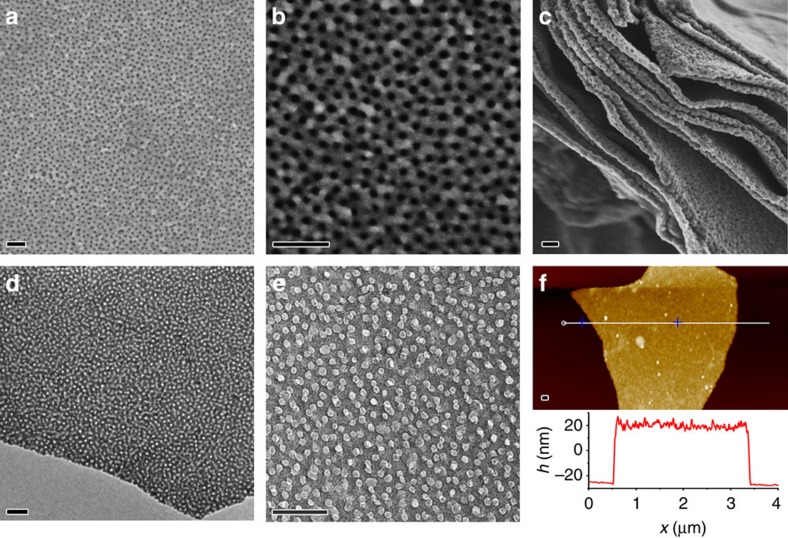
Structural characterization of 2D GO-based mesoporous PPy nanosheets. (**a**–**c**) SEM images of mesoporous PPy nanosheets synthesized with the PS_102_-*b*-PEO_114_, demonstrating the highly regular large-pore mesoporous structures. (**d**,**e**) TEM images of the samples. (**f**) AFM survey of the sample (Scale bar, 100 nm).

Figure 2 shows the structural characterization of the GO-based mesoporous PPy nanosheets synthesized from PS_102_-*b*-PEO_114_ (mPPy@GO-2) after the removal of BCP templates. Template removal is demonstrated by the disappearance of characteristic peaks of PEO and PS blocks in the Fourier-transform infrared and solid-state NMR spectroscopy (Supplementary Figs 4 and 5). Scanning electron micrographs (SEMs) reveal that the PPy nanosheets possess a partially ordered pore array with the pore size of ∼13 nm, which is in accordance with the TEM results in Fig. 2d,e. The partial overlap of mesopores on both GO surfaces observed from TEM images (Fig. 2e) confirms that the mesopores lie on both sides of the GO, consistent with SEM side view on the PPy nanosheets (Fig. 2c), indicating that the growth of mesoporous PPy nanosheets was directed by adaptation of the GO template sheet morphology into a sandwich structure. The AFM image in Fig. 2f reveals that thin-layer mPPy@GO nanosheets have a flat surface, a uniform height of ∼45 nm, and a thickness almost equal to twice the diameter of PS_102_-*b*-PEO_114_ micelles. This result supports the confined growth mechanism of PPy on both sides of the GO surface. The control experiments demonstrate the importance of the choice of structure-directing BCP templates and 2D GO surface for achieving such unique architectures. As shown in Supplementary Fig. 6, under the same conditions, only irregular PPy nanoparticles were obtained when the BCP template and GO dispersion were not added, while the growth of PPy on the GO surface without the use of BCP micelles resulted in smooth PPy nanosheets without any mesoporous structure.

### Controlled Patterning of 2D free-standing surfaces

**Figure 3:**
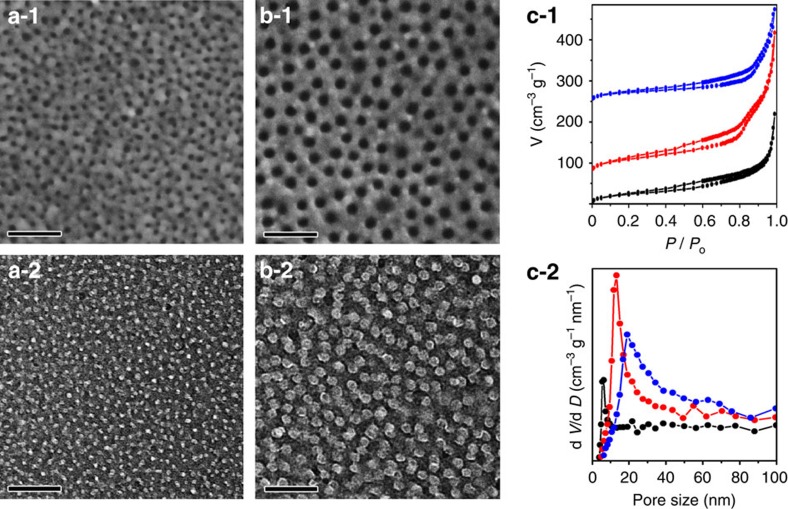
Patterning of 2D free-standing surfaces with controlled mesopores. (**a**) SEM image (**a**-**1**) and TEM image (**a**-**2**) of mPPy@GO-1 nanosheets. (**b**) SEM image (**b**-**1**) and TEM image (**b**-**2**) of mPPy@GO-2 nanosheets. (**c**) N_2_ adsorption/desorption isotherms of (**c**-**1**) mPPy@GO nanosheets (black: mPPy@GO-1, red: mPPy@GO-2 and blue: mPPy@GO-3), where the red and blue isotherms are vertically offset by 75 and 250 cm^3^ g^−1^ STP, respectively. (**c**-**2**) The pore size distribution curves calculated from the adsorption branches (scale bar, 100 nm).

By simply changing the PS block lengths, the patterning of conducting polymers on 2D free-standing surfaces with various pore sizes and thickness can be realized. When using the PS_38_-*b*-PEO_114_ template with a shorter PS block, the pore size decreases to below 6 nm with the thickness of ∼35 nm (Fig. 3a; Supplementary Fig. 9). When the PS block length is increased to a degree of polymerization of 146, the pore size of mPPy@GO-3 nanosheets increased to ∼20 nm (Fig. 3b) and the thickness increased to ∼44 nm. In addition, the pore–pore distance of mPPy@GO-1 nanosheets is ∼13.5 nm, while that of mPPy@GO-3 nanosheets is ∼17.4 nm, indicating that the pore–pore distances of mPPy@GO are more dependent on the hydrophilic PEO length than on the size of the PS block.

Brunauer–Emmett–Teller (BET) measurements shine further light on the pore features of mPPy@GO nanosheets. N_2_ sorption isotherms (Fig. 3c–1) show a type IV curve with a H_2_-type hysteresis loop. The adsorption step associated with capillary condensation gradually shifts to higher pressures with increased PS chain length, indicating an increase in the mesopore size. The BET surface area and pore volume of mPPy@GO-2 nanosheets are calculated to be 85 and 0.29 cm^3^ g^−1^ (Supplementary Fig. 10; Supplementary Table 2), respectively. Despite having the larger pore size and pore–pore distances, the surface area of the mPPy@GO nanosheets are still 2.8–3.5 times larger than that of PPy@GO nanosheets synthesized without any BCP templates. The PSD curves (Fig. 3c–2) indicate that mPPy@GO-1, mPPy@GO-2 and mPPy@GO-3 nanosheets are characterized by uniform pore sizes of 5.8, 13.2 and 19.3 nm, respectively, which is consistent with SEM and TEM observations. After carbonization at 550 °C, the specific surface areas of the mPPy@GO-2 nanosheets could be increased up to 165 m^2^ g^−1^. The mesoporous structure and pore size of the pyrolyzed mPPy@GO nanosheets have no obvious change compared with the as-made samples, even after treatment at 850 °C (Supplementary Figs 10 and 11; Supplementary Table. 2), indicating the high stability of the mesoporous structure of mPPy@GO nanosheets.

### Direct patterning of various free-standing functional surfaces

**Figure 4:**
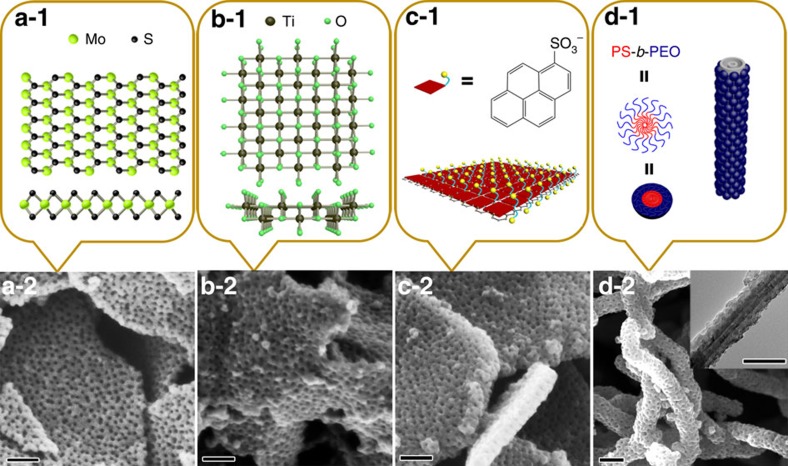
Patterning of various functional free-standing surfaces. (**a-1**) Structure of MoS_2_ nanosheets in top view (upper) and side view (lower). (**a-2**) SEM image of large-pore mesoporous PPy nanosheets on MoS_2_ nanosheets. (**b-1**) Structure of titania nanosheets in the [010] (upper) and [001] (lower) directions. (**b-2**) SEM image of large-pore mesoporous PPy nanosheets on titania nanosheets. (**c-1**) Illustration of the 1-PSA-modified EG surfaces. (**c-2**) SEM image of large-pore mesoporous PPy nanosheets on EG nanosheets following modification with 1-PSA. (**d-1**) Illustration of CNTs wrapped by PS-*b*-PEO micelles. (**d-2**) SEM image of large-pore mesoporous PPy nanosheets on CNTs (inset of **d** is TEM image; scale bar: 100 nm).

This BCP-directed approach can be further adapted for the direct patterning of mesoporous conducting polymers on other functional surfaces. Taking the 2D free-standing nanosheets of MoS_2_, titania and electrochemically EG as examples, a series of mesoporous conducting nanosheets with similar sandwich structures can be obtained. Figure 4a shows the large-pore mesoporous structures and sandwich morphologies that result from patterning PPy on the surface of ultrathin MoS_2_, which was exfoliated using the lithium ion intercalation method^39^. Similarly, using ultrathin titania as the 2D free-standing surface and PS_102_-*b*-PEO_114_ as the template, titania-based mesoporous PPy nanosheets with regular pore arrays and large pore sizes of ∼12 nm can also be obtained (Fig. 4b). Similar to the GO system, BCP micelles can be also effectively adsorbed onto both surfaces of MoS_2_ and titania nanosheets via H-bonding interaction (Supplementary Fig. 3), which further guides the confined adsorption of pyrrole monomers onto the negatively charged surfaces of MoS_2_ and titania, and results into the successful patterning of PPy in 2D manner.

Patterning conducting polymers on electrochemically EG is another appealing objective due to the high electrical conductivity and pristine nature of graphene compared with that of GO. However, the lack of available oxygen functional groups on the EG surfaces results in poor dispersibility in aqueous solution. Thus, it is very difficult for EG surfaces to directly interact with BCP micelles in aqueous solution. To overcome this obstacle, a surfactant, 1-pyrenesulfonic acid sodium salt (1-PSA), was introduced to functionalize the EG surface, as illustrated in Fig. 4c–1. In this respect, the pyrene moiety in 1-PSA can effectively interact with EG via strong π–π interactions^40^, while the exposed sulfonyl group provides negative charges to EG (zeta potential of approximately −45.2 mV) to promote dispersion in aqueous solution and induce interactions with BCP micelles and monomers via H-bonding. Thus, with the assistance of the 1-PSA, mPPy@EG nanosheets with well-defined sandwich morphology and large-pore mesopores are achieved (Fig. 4c). The pore size of mPPy@EG nanosheets is ∼12 nm, consistent with that of the above mesoporous PPy nanosheets with PS_102_-*b*-PEO_114_. The thickness (∼50 nm) of mPPy@EG is slightly larger than that of the PPy nanosheets, which is attributed to the increased number of EG layers that occurs due to electrochemical exfoliation and the non-covalent functionalization with 1-PSA.

In addition to the above-described 2D surfaces, we found that this method can also be utilized for 1D multiwall CNTs to produce unique mesoporous core–shell cable structures. Here the functionalized CNTs by treatment with HNO_3_ possess the abundant oxygen-containing groups (such as –COOH and –OH), similar to the GO surfaces. SEM images in Fig. 4d clearly display 1D architectures with mesoporous PPy nanoshell-encapsulated CNTs. The pore size of these mesoporous PPy nanoshells is ∼12 nm, consistent with that of the sandwich-structured mPPy@GO-2 nanosheets prepared with BCP templates.

Our strategy can be further utilized to pattern PANi, another highly promising conducting polymer due to its chemical stability, fast redox rate and doping–dedoping process, on various 2D surfaces. Compared with PPy, the presence of a primary amine in PANi improves electrostatic adsorption and self-assembly on negatively charged 2D surfaces. Supplementary Fig. 12 shows that a series of large-pore mesoporous PANi nanosheets have been successfully patterned on various 2D free-standing surfaces, including EG, GO, MoS_2_ and titania nanosheets. These results clearly demonstrate that our versatile strategy is a powerful tool to construct 2D mesoporous nanosheets with controlled pore sizes on functional surfaces.

### Electrochemical performance of mesoporous hybrid nanosheets

**Figure 5:**
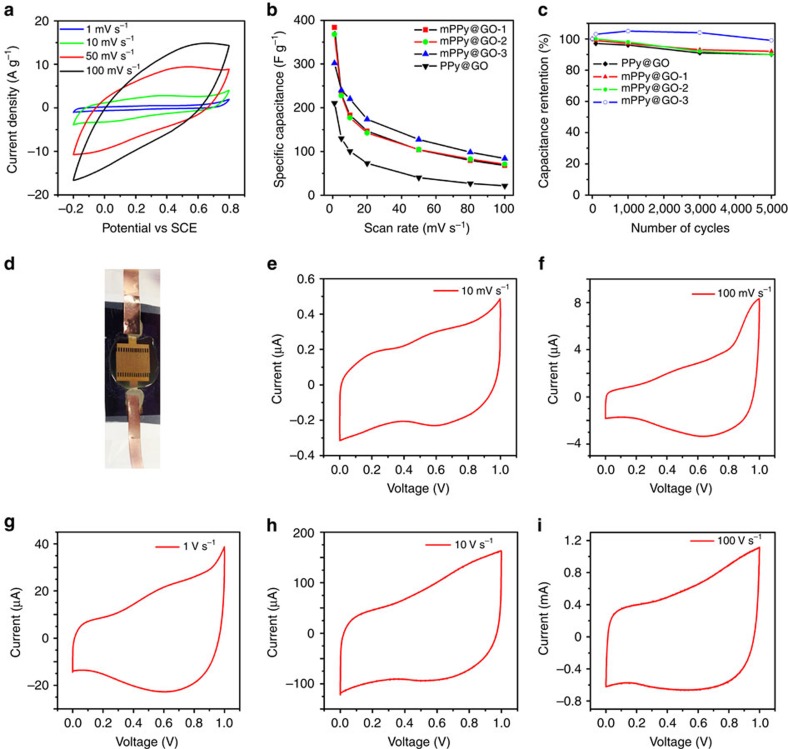
Electrochemical performance of 2D large-pore mesoporous mPPy@GO nanosheets. (**a**) CV curves of mPPy@GO nanosheets synthesized by PS_38_-*b*-PEO_114_ as electrodes at different scan rates, (**b**) comparison of specific capacitance versus scan rate for mPPy@GO-1 (5.8 nm), mPPy@GO-2 (13.2 nm), mPPy@GO-3 (19.2 nm) and mPPy@GO nanosheet electrode materials and (**c**) Electrochemical cycling stability of mPPy@GO-1, 2 and 3 and mPPy@GO nanosheet electrode materials at a high scan rate of 50 mV s^−1^. (**d**) Photograph of the on-chip all solid-state mPPy@GO micro-supercapacitors with in-plane geometry. (**e**–**i**) CV curves of mPPy@GO-3 nanosheets as electrodes for micro-supercapacitor at scan rates from 0.01 to 100 V s^−1^.

Due to their unique sandwich structure and regular mesoporous array, the obtained mesoporous nanosheets are expected to serve as a novel class of promising electrode materials for various electrochemical applications. As a proof of concept, we evaluated the electrochemical properties of the synthesized GO-based mesoporous PPy nanosheets (mPPy@GO) with mesopore sizes of 5.8, 13.2 and 19.3 nm (denoted as mPPy@GO-1, mPPy@GO-2 and mPPy@GO-3, respectively) as electrodes in supercapacitors. Their performance was compared with that of PPy@GO nanosheets without mesoporous structures (Supplementary Fig. 6c). Figure 5a presents the cyclic voltammetry (CV) curves of the mPPy@GO-1 nanosheets. It is clear that the CV curves exhibit the typical pseudocapacitive behaviour of PPy with strong redox peaks in the range of −0.2 to 0.8 V at low scan rates of 1 mV s^−1^. Similar results were also observed for mPPy@GO-2 and mPPy@GO-3. At the same scan rates, all of the tested mesoporous mPPy@GO nanosheets exhibit much higher current densities and CV curve areas than that of the PPy@GO nanosheets (Fig. 5; Supplementary Fig. 13), suggesting that the presence of mesopores in mPPy@GO nanosheets would be extremely beneficial for the enhancement of the electrochemically capacitive behaviour.

Figure 5b shows the capacitance and rate performance of mPPy@GO and PPy@GO nanosheets. Notably, the mPPy@GO-1 nanosheets with small mesopores of 5.8 nm deliver an impressive specific capacitance of 383 F g^−1^ at 1 mV s^−1^, which is higher than those of the large mesopore mPPy@GO-2 (368 F g^−1^) and mPPy@GO-3 (302 F g^−1^), and superior to most of the state-of-the-art results of graphene-based PPy electrode materials (Supplementary Table 3)^41^,^42^,^43^. In addition, all mPPy@GO nanosheets exhibit much higher specific capacitance than the PPy@GO nanosheets (201 F g^−1^), implying that the mesoporous structure contributes to the fast ion diffusion and high-charge storage behaviour. Especially, at a high scan rate of 100 mV s^−1^, the specific capacitance of all mPPy@GO nanosheets (mPPy@GO-1: 68 F g^−1^; mPPy@GO-2: 72 F g^−1^ and mPPy@GO-3: 85 F g^−1^) present three times greater than that of the PPy@GO nanosheets (22 F g^−1^). Moreover, mPPy@GO-3 with larger mesopore size and initial capacitance retention of 27.8% shows better rate capability than mPPy@GO-1 (17.8%) and mPPy@GO-2 (19.3%), suggesting that larger porous channels are more favourable for rapid accessibility of ions from the exterior electrolyte to the interior electrode, leading to high rate capacitance^42^. Moreover, all of the 2D nanosheet electrodes exhibit excellent cycling stability with above 90% capacitance retention after 5,000 cycles at a scan rate of 50 mV s^−1^ (Fig. 5c). Remarkably, the mPPy@GO-3 electrode in comparison with others can maintain ∼99% of the initial capacitance after long-term cycling, indicating that larger mesopores in such unique 2D architecture can well accommodate large volumetric expansion and counterion drain effect during rapid charge and discharge process^44^. In addition, the initial enhanced capacity electrode can be attributed to the improvement of ion accessibility in 2D mesopores nanosheets during the long charge and discharge process, resulting in an increased accommodation behaviour for charges, and the similar phenomenon has been observed in the previous reports^45^,^46^.

To further evaluate their electrochemical performance, we fabricated the on-chip all-solid-state planar MSCs (denoted as mPPy@GO-MSCs) based on the mPPy@GO-3 film with a thickness of around 2 μm on silicon wafer, using H_2_SO_4_/polyvinyl alcohol gel electrolyte (Fig. 5d). The electrochemical performance of mPPy@GO-MSCs was studied by CV (Fig. 5e–i). Clearly, mPPy@GO-MSCs showed a remarkable redox response at different scan rate ranging from 10 mVs^−1^ to 100 V s^−1^, suggestive of strong pseudocapacitive characteristics from the PPy contribution. Notably, at a high scan rate of 100 V s^−1^, corresponding to an extremely short discharging time of only 10 ms, mPPy@GO-MSCs still delivered an impressive areal capacitance of 19 μF cm^−2^, maintaining over 25% of the initial capacitance (75.5 μF cm^−2^ at scan rate of 10 mV s^−1^). This result demonstrates the high capacitance and rate capability of mPPy@GO-MSCs.

## Discussion

In summary, we have demonstrated a robust and versatile protocol for controlled patterning of mesoporous conducting polymers of PPy and PANi on various functional free-standing surfaces via solution-based assembly of close-packing monolayers of BCP micelles, including 2D electrochemically EG, GO, MoS_2_ and titania nanosheets as well as 1D CNTs. This patterning technique allows for successful production of a series of 2D ultrathin hybrid materials with unique sandwich structure, adjustable pore sizes and thickness. Such novel structure leads to an enhanced electrochemical capacitance and rate performance when applied as supercapacitor and MSC electrodes. Furthermore, given the diversity of available BCP templates and strong interactions with functional surfaces and precursors (such as metal, oxides, organic polymer and so on), this modular approach would offer a new direction towards the controlled synthesis of 2D hybrid materials with well-defined mesoporous architectures and potential applications in a wide range of areas.

## Methods

### Patterning of mesoporous PPy nanosheets onto GO

In a typical experimental procedure, 0.05 g of PS_102_-*b*-PEO_114_ BCP was dissolved in 1 ml THF. Then, 1 ml of deionized (DI) water was slowly added to the solution to generate the micellar aggregation. The solution was added to 7 ml DI water to quench aggregation. After stirring, 0.75 ml of GO solution (2 mg ml^−1^), which was synthesized by a modified Hummers method^47^, and 31 μl of pyrrole were injected into the solution. Then, 0.09 g of ammonium persulfate was used to initiate the polymerization of pyrrole monomers into PPy. The 2D mPPy@GO nanosheets were obtained after repeatedly washing the BCPs and excess ions with THF and water. The same amount of BCP templates with different number of styrene units of 38, 102 and 146 was utilized to synthesize mPPy@GO nanosheets with tunable pore sizes.

### Patterning of mesoporous PPy nanosheets on MoS_2_ and titania

First, MoS_2_ monolayers were prepared by lithium intercalation and further ultrasonication exfoliation^39^. The concentration of the exfoliated MoS_2_ dispersion was ∼0.4 mg ml^−1^. The synthesis method of mPPy@MoS_2_ was identical to that of mPPy@GO except for the amount of MoS_2_, which was 12 ml. Titania nanosheets were synthesized according to the literature yielding a concentration of ∼4 mg ml^−1^ (ref. 48). For each sample of mPPy@TiO_2_, 6 ml titania solution was used and the other procedures were the same as for the other samples of mPPy@GO and mPPy@MoS_2_.

### Patterning of mesoporous PPy and PANi nanosheets on electrochemically EG

*Modification of EG with 1-PSA*. Electrochemically EG was prepared according to a previous report^34^. After exfoliation, 0.12 g EG in 50 ml *N,N*-dimethylmethanamide solution was dispersed with the surfactant molecule 1-PSA. A amount of 0.10 g 1-PSA was added to the above solution and ultrasonicated for 30 min. After centrifugation at 14,000 r.p.m. min^−1^, the supernatant was discarded. Then, 0.10 g 1-PSA and DI water were added and ultrasonicated for further 30 min. After centrifugation and removal of the supernatant, the final product was dispersed in DI water, which yielded a concentration of 1.1 mg ml^−1^ for EG.

*Synthesis of mPPy@EG and mPANi@EG*. Typically, 0.05 g PS_102_-*b*-PEO_114_ was dissolved in 1 ml THF, then 1 ml DI water was slowly added to the solution. This mixture was added to 7 ml DI water. After stirring for a while, 6 ml EG modified with 1-PSA solution and 31 μl pyrrole were injected into the above solution. Afterwards, 0.09 g ammonium persulfate was added for initiating the polymerization of pyrrole monomers into PPy. The mPPy@EG nanosheets were obtained after repeated washing with THF and water to remove the BCPs and excess ions. For the synthesis of mPANi@EG, 35 μl aniline was injected into the above solution. Then, 1 g HCl (1 mol l^−1^) was added to the above solution and 0.09 g ammonium persulfate was used to polymerize aniline monomers.

*Synthesis of mesoporous PPy@CNT cables*. A amount of 0.05 g PS_102_-*b*-PEO_114_ was first dissolved in THF and 1 ml DI water was slowly added to the solution and the mixture was introduced into 7 ml DI water. After stirring for a while, 2 ml CNTs solution (6 mg ml^−1^, treated with concentration of HNO_3_ at 100 °C for 16 h) and 31 μl pyrrole were injected into the above solution. Afterwards, 0.09 g ammonium persulfate was added for initiating the polymerization of pyrrole monomers. Finally, the mesoporous PPy@CNTs nanocables were obtained after repeated washing steps with THF and water to remove the BCPs and ions.

*Patterning of mesoporous PANi nanosheets on GO, MoS_2_ and titania*. In a typical experiment procedure, 0.05 g PS_102_-*b*-PEO_114_ was dissolved in 1 ml THF. Then, 1 ml DI water was slowly added to the solution. The resulting mixture was introduced into 7 ml DI water. After stirring for a while, 0.75 ml GO solution (2 mg ml^−1^) and 35 μl aniline were injected into BCP solution. Afterwards, the dispersion was acidified with 1 g HCl (1 mol l^−1^) and 0.09 g ammonium persulfate was added to polymerize aniline monomers. Finally, the 2D mPANi@GO nanosheets were obtained after repeated washing with THF and water to remove the BCPs and excess ions. As for the synthesis of mPANi@MoS_2_ and mPANi@titania, the amount of MoS_2_ and titania were 12 and 6 ml, respectively, while the other conditions remained.

### Characterization of 2D mesoporous hybrid materials

The morphology of the samples was examined with a Gemini 1530 (Carl Zeiss AG, Oberkochem, Germany) SEM operated at 1 kV and a TEM (JEOL-1400) operated at an accelerating voltage of 120 kV. Powder XRD patterns were recorded on a Rigaku X-ray diffractometer D/MAX–2200/PC equipped with Cu–Ka radiation (40 kV, 20 mA) at a rate of 1.0° min^−1^ over the range 5–80 (2*θ*). X-ray photoemission spectroscopy measurements were performed on a PHI**–**5000C ESCA system with a monochromatic Mg Kα X-ray source (*h* × *v*=1,253.6 eV), the C 1*s* value was set at 284.6 eV for charge corrections. Raman spectra were recorded with a Bruker RFS 100/S spectrometer (laser wavelength 532 nm). Fourier-transform infrared spectra between 4,000 and 400 cm^−1^ were recorded using a Nicolet iS10. Nitrogen sorption isotherms were measured at 77 K on ASAP 2020 and TriStar 3020 volumetric analyzers (Micromeritics, nc, GA). Before measurements, all samples were degassed at 120 °C for at least 12 h. Specific surface area was determined by standard BET method in the relative pressure range of 0.05–0.2 *P*/*P*_o_. The PSD curves were calculated by the Barrett–Joyner–Halenda method using the adsorption branch of the isotherm. The hydrodynamic diameters *D*_h_ of BCP micelles were determined with a Nicomp TM 380 Submicron Particle Sizer (PSS-Nicomp) at an angle of 90°. Gel permeation chromatography was performed on Agilent Technologies 1260 Infinity instrument using THF as eluent calibration was performed with polystyrene standards. AFM images were recorded in air on a Veeco Dimension 3100. To directly observe the self-assembly of BCP micelles in aqueous solution, AFM images were recorded as detailed below. Solid-state NMR 1H MAS as well as 13C (1H) MAS 1D measurements have been performed on Bruker 500 MHz Avance III system with a spinning frequency of 25 kHz. A sweep width of 100 kHz for proton (2.5 μs *π*/2 pulse) and 50 kHz for carbon (1 μs *π*/5 pulse) was used. All experiments were performed double-resonance H-X probe for rotors with 2.5 mm outside diameter. The high-power decoupling 13C (1H) MAS 1D measurements were pre-optimized using L-alanine and for the 1H MAS 1D adamantine was utilized at 298 K. The mean *Z*-potential (mV) was determined with a MALVERN Zetasizer Nano ZS (Malvern Instruments Ltd, United Kingdom) by performing electrophoretic experiments on the samples and measuring the particle’s velocity by the technique of laser Doppler velocimetry. The measurements were carried out on a glass dip-cell at 25 °C and an apply voltage of 5 V for most samples. To preserve the existing charge state of the surfaces, the measurements were performed on the same media as the synthesis: a (H_2_O:THF) solution with a volume ratio of 8:1, which can be considered as mostly aqueous, and therefore the Smoluchowski model can be applied to analyse the data.

### AFM characterization of assembly of BCP micelles in aqueous solution

A 100-μl GO dispersion was spin coated on a Si/SiO_2_ surface with a size of 1 by 1 cm and spinning speed of 500 r.p.m. A drop of 50 μl freshly prepared micelle solution was drop casted on the middle of the GO film. Then, the micelles were allowed to immobilize for 20 min in a Petri dish followed by intensive washing employing 0.5 ml MQ water. Afterwards, the sample was immediately placed into a liquid cell and additional 100 μl MQ water was injected inside the AFM fluid cell. During the whole measurement, the sample was kept in wet conditions. Next, the fluid cell with the sample was kept for 3–6 h for liquid stabilization and for the equilibrating the temperature of AFM cantilever and the water. The scanning measurements were performed with an AFM Multimode 8, Controller V in tapping mode. The sharp nitrate level-A (SNL-A) probes were exhibited a spring constant of 0.35 N m^−1^, a resonant frequency of 7.3 kHz (in aqueous media) and a tip radius ∼2 nm. The AFM height images were recorded over regions of 10–30 μm with resolution of 2,048–3,072 lines per sample with a scanning speed of 0.2 Hz. This corresponds to recording times of up to 2 h per image. During sample imaging, the taping force was chosen manually as low as possible to minimize deformation of the samples and to prevent ‘expelling’ of micelles from the self-assembled arrays by the AFM probe. The non-distracting method of the scanning was clarified by recording trace and retrace pathways for the same scanning area. The height profiles of the images were analysed using NanoScope Analysis software.

### Electrochemical measurements

*Three-electrode test system*. The working electrode was prepared by mixing 80 wt% mPPy@GO, 10 wt% carbon black and 10 wt% poly(tetrafluoroethylene) binder dispersed in ethanol, and then pressing the onto a platinum mesh (1 × 1 cm) as current collector. The electrochemical capacitive of mPPy@GO and PPy@GO was evaluated in a three-electrode system, applying 1 M H_2_SO_4_ as electrolyte, platinum plate (1 × 2 cm) and Ag/AgCl (saturated KCl) as the counter and reference electrodes, respectively. The CV and galvanostatic charge–discharge measurements were performed on a CHI 760D electrochemical workstation with a potential range from −0.2 to 0.8 V.

*MSC test system*. The two-electrode test has been used to evaluate the performance of the on-chip solid planar MSCs. To fabricate MSCs, as synthesized PPy@GO was dispersed in dimethylformamide, followed by a filtration-based dry transfer method, a PPy@GO film on SiO_2_/Si substrate was obtained. After fully evaporation of the solvent residue, 30 nm gold (Premion, 99.9985% metals basis, Alfa Aesar) was thermally evaporated (EDWARDS FL400) on the PPy@GO film with a rate of ∼1.0 Å s^−1^ and a chamber pressure of ∼1 × 10^−6^ torr through a home-made 30-interdigital finger mask (widths of 210 μm and interspaces of 70 μm). Then, the electrode micro-patterns of PPy@GO film on the substrates were created by oxidative etching of the exposed film in an O_2_-plasma cleaner with 20 s.c.c.m. O_2_ flow for several minutes and 100–200 W radio frequency power (Plasma System 200-G, Technics Plamsa GmbH), the etching depth was step-by-step inspected by digital Multimeter. Afterwards, H_2_SO_4_/poly(vinyl alcohol) (PVA) gel electrolyte was slowly drop casted onto the surface of interdigital electrodes and solidified overnight. Finally, PPy@GO film-based in-plane MSCs was achieved. The gel electrolyte was prepared by mixing 6 g PVA (molecular weight=89,000–98,000, Sigma-Aldrich) and 6 g H_2_SO_4_ in 60 ml DI water, and was heated at 85 °C for 1 h under stirring. CV measured at the different scan rates ranging from 0.01 to 100 V s^−1^, and galvanostatic charge and discharge profiles at various current density, was carried out by a CHI 760D electrochemical workstation. All electrochemical experiments were carried out at room temperature.

## Additional information

**How to cite this article:** Liu, S. *et al*. Patterning two-dimensional free-standing surfaces with mesoporous conducting polymers. *Nat. Commun.* 6:8817 doi: 10.1038/ncomms9817 (2015).

## Supplementary Material

Supplementary Information Supplementary Figures 1-15, Supplementary Tables 1-3 and Supplementary References
