# Supplementary material for: Patterning two-dimensional free-standing surfaces with mesoporous conducting polymers
Source: Nat Commun. 2015 Nov 18;6:8817. doi: 10.1038/ncomms9817 (PMC4660032; doi:10.1038/ncomms9817)
Supplement: Supplementary Information — Supplementary Figures 1-15, Supplementary Tables 1-3 and Supplementary References [file ncomms9817-s1.pdf]

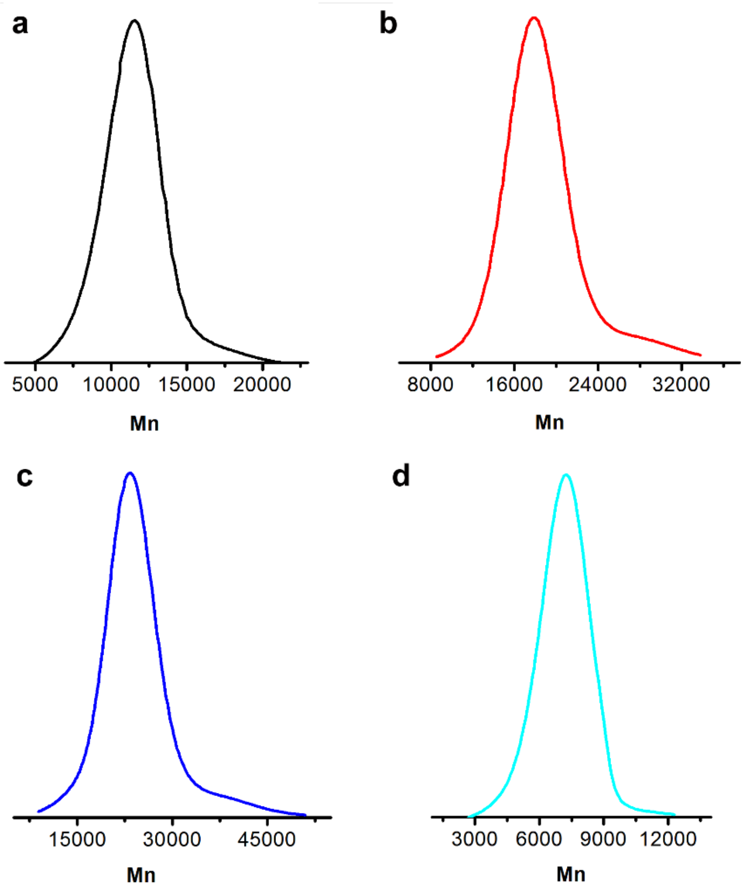

Supplementary Figure 1. Gel permeation chromatography (GPC) traces of PS_{38}-*b*-PEO_{114} (**a**), PS_{102}-*b*-PEO_{114} (**b**), PS_{146}-*b*-PEO_{114} (**c**) and PEO_{114}-Br (**d**), which show narrow molecular-weight distributions with polydispersity index (PDI) of 1.05, 1.04, 1.06 and 1.04, respectively.

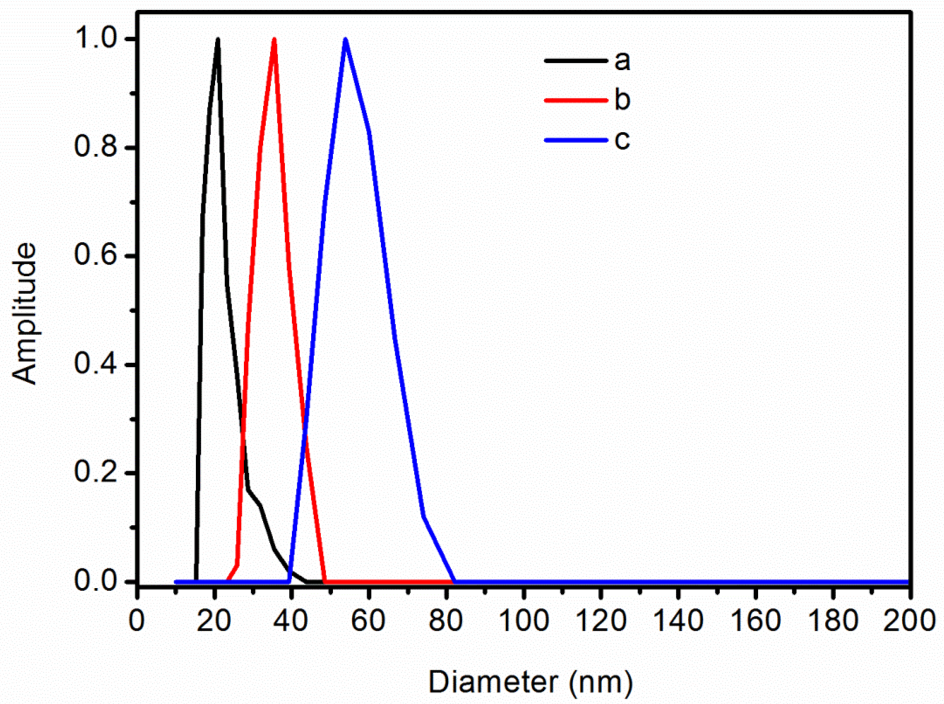

Supplementary Figure 2. The hydrodynamic diameters D_h of BCP micelles of PS_{38}-*b*-PEO_{114} (**a**), PS_{102}-*b*-PEO_{114} (**b**) and PS_{146}-*b*-PEO_{114} (**c**), showing that micelles exhibit narrow particle size distributions with diameters of ~21 nm, ~35 nm and ~54 nm, respectively.

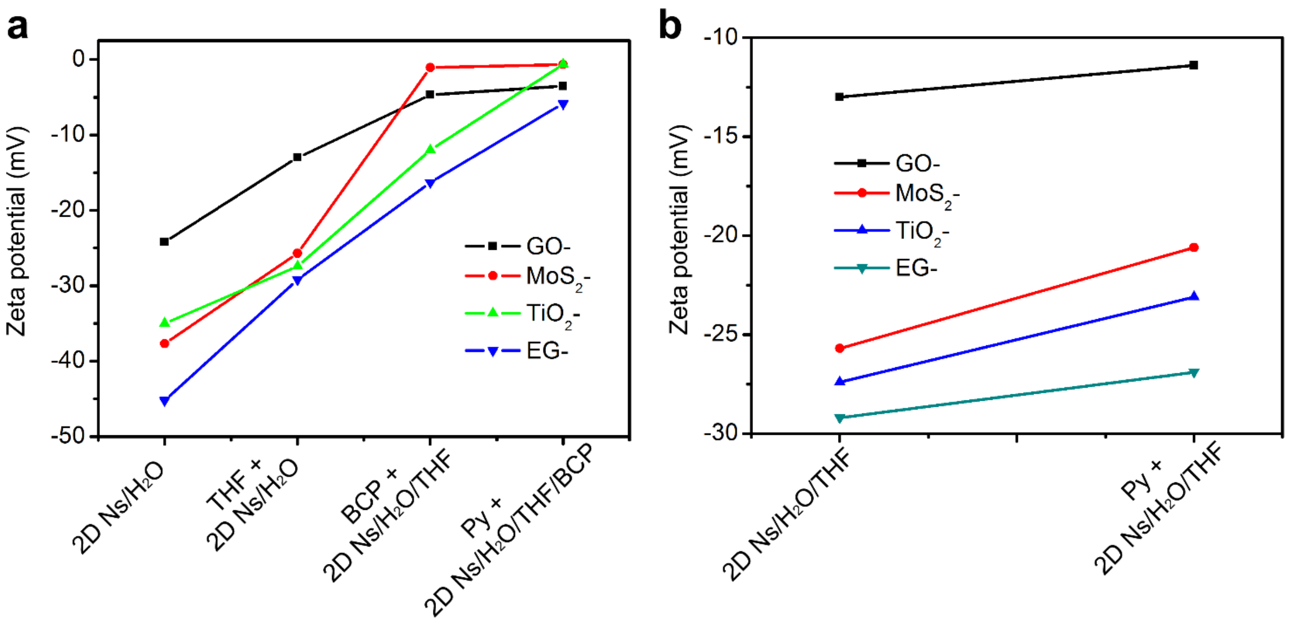

Supplementary Figure 3. Zeta potentials of 2D nanosheets (2D Ns) in aqueous solution and the mixture of THF/H_2O (1/8, v/v), and the change of Zeta potentials after adding BCP and pyrrole monomer (**a**); Zeta potentials of 2D nanosheets (2D Ns) in the mixture of THF/H_2O (1/8, v/v) and their change after adding pyrrole monomer (**b**).

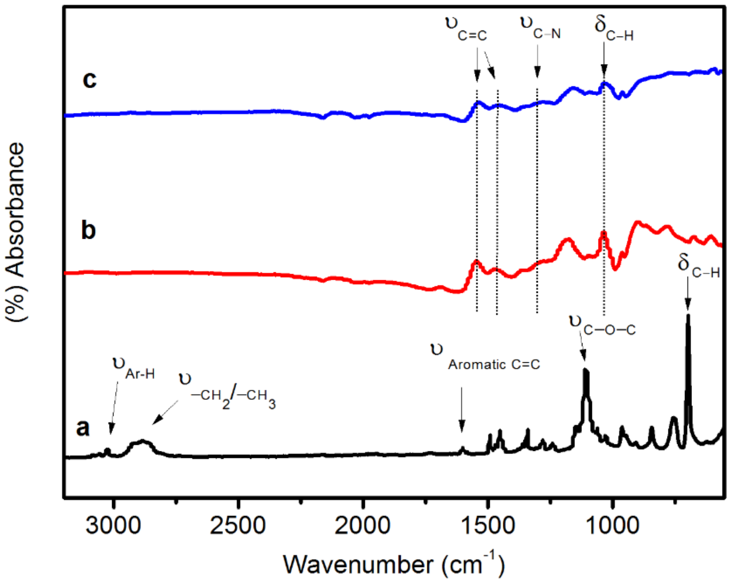

Supplementary Figure 4. FT-IR spectra of the block copolymer PS_{102}-*b*-PEO_{114} (**a**), the as-made mesoporous PPy nanosheets on GO synthesized by block copolymer PS_{102}-*b*-PEO_{114} (**b**) and the as-made PPy nanosheets on GO synthesized without block copolymer template (**c**). For curve a, the bands at 1100 cm^{-1} are associated with PEO block, while bands at 2850-2930 cm^{-1}, 1602 and 700 cm^{-1} are corresponding to PS block. The disappearance of characteristic peaks of PEO and PS in spectrum b suggests the removal of PEO-*b*-PS template by extraction using THF. Moreover, the characteristic stretching vibration bands of pyrrole rings at 1549 and 1470 cm^{-1}, and the bands characteristic for stretching of conjugated C-N and planar C-H at 1302 and 1042 cm^{-1}, respectively, demonstrate the formation of PPy.

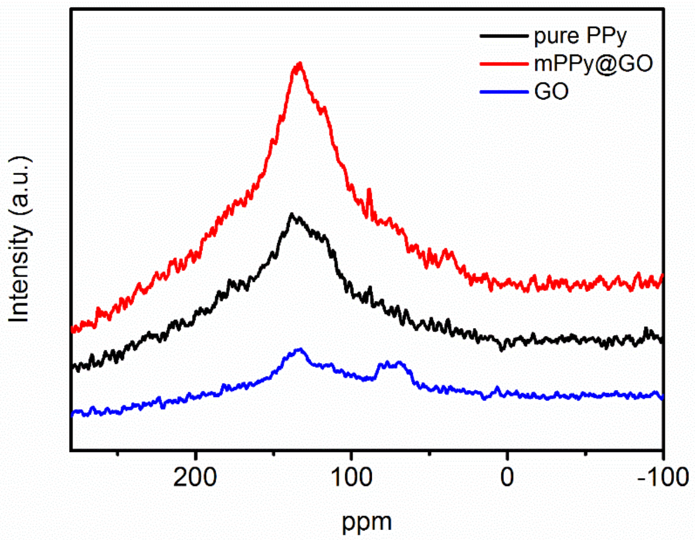

Supplementary Figure 5. Solid-state ^{13}C nuclear magnetic resonance (NMR) spectra of GO, pure PPy and mPPy@GO samples. ^{13}C solid state MAS NMR 1D measurements have been performed to further verify the complete removal of BCP templates from the mPPy@GO. The signals around 60~80 ppm are assigned to C-O-C expoxide groups and C-OH groups, and the signals at 112~133 ppm are assigned to conjugated double bonds in aliphatic rings and aromatic entities.[1] For the mPPy@GO samples, those typical signals of GO have been replaced by the abroad signals at 117~140 ppm because of the coating of PPy.[2] Additionally, the curve of mPPy@GO samples is almost the same with that of pure PPy, as well as no obvious signal has been found at 20.6 ppm for methyl carbon and at 70 ppm for EO units indicate the successful removal of BCP templates for mPPy@GO samples.

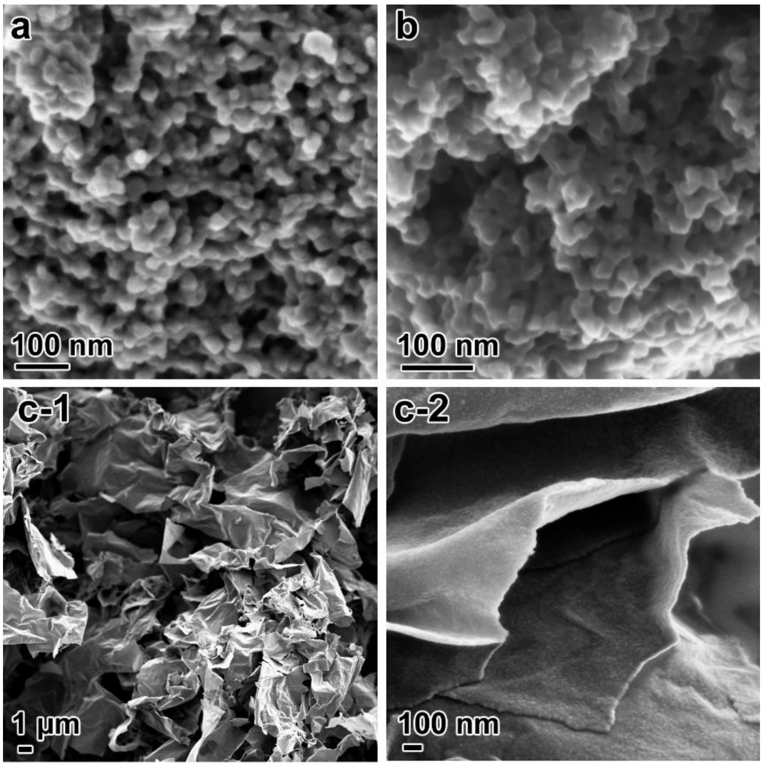

Supplementary Figure 6. SEM images of PPy synthesized as control experiments under the same conditions as for mPPy@GO. (**a**) PPy nanoparticles synthesized without GO and BCP templates. (**b**) PPy nanobowls synthesized without GO after adding BCP templates. (**c**) PPy@GO nanosheets synthesized without BCP templates.

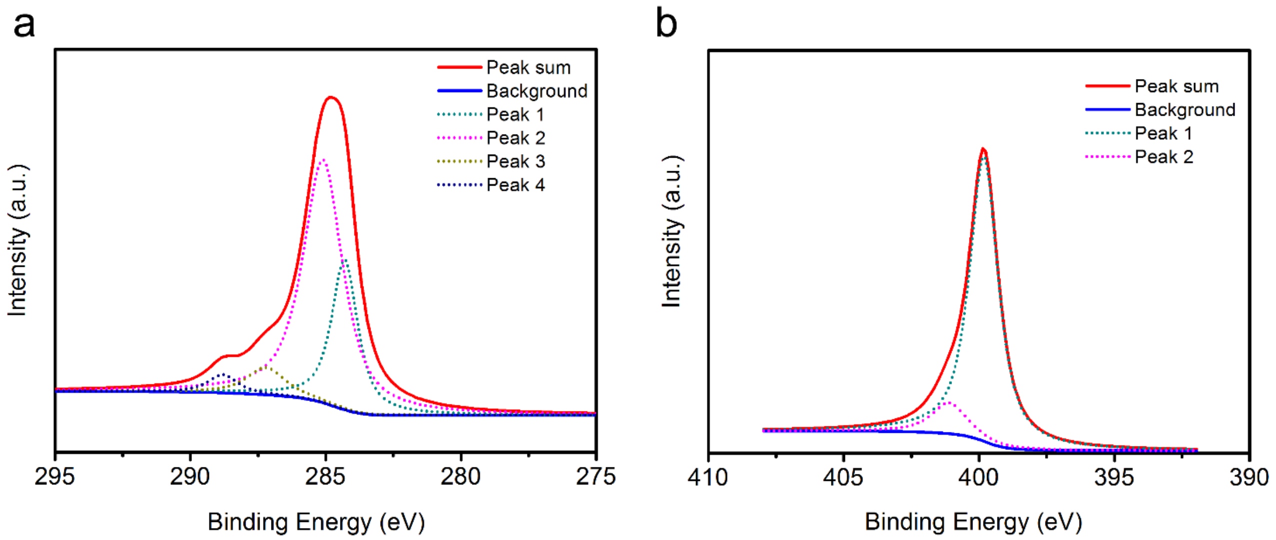

Supplementary Figure 7. C1s (**a**) and N1s (**b**) X-ray photoelectron spectroscopy (XPS) core level spectra of synthesized mPPy@GO. The enhanced intensity of the *α*-carbon peak (peak-2, corresponding to 285.1 eV) compared with the other peaks, implies the predominant *a-a'* bonding.[3,4] The small area of the high-binding-energy wings of the C1s spectra (*i.e.* at ~288 eV peak) indicates that the synthesized mPPy@GO materials contain low defects.[5] Additionally, N1s core level spectra show the lower area ratio (13.3%) of the peaks at ~401.1 eV and ~399.8 eV peaks, suggesting the lower dopant level.[6]

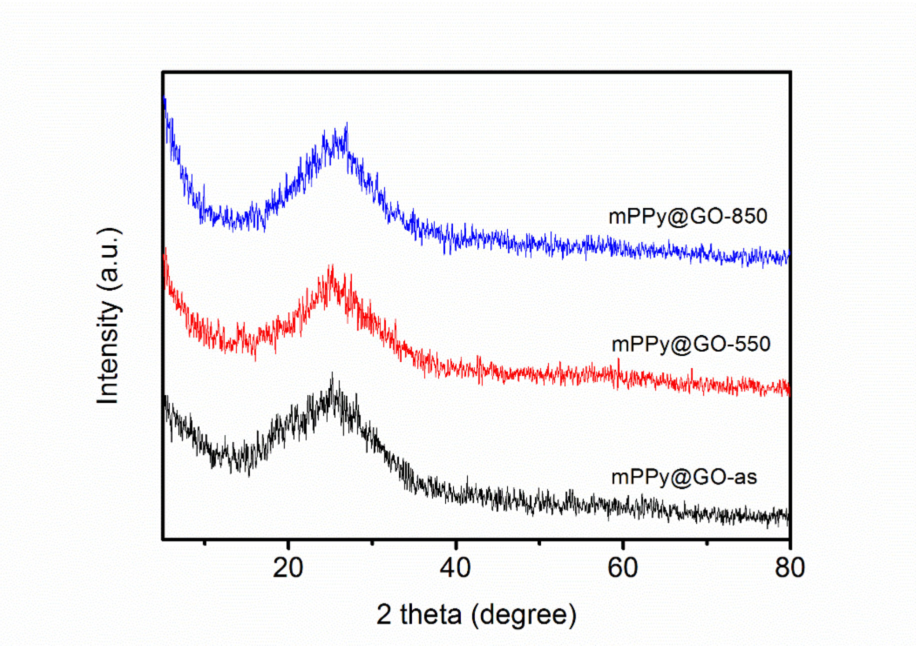

Supplementary Figure 8. X-ray diffraction (XRD) curves of the mPPy@GO nanosheets and the carbonized samples. As-made mPPy@GO shows only a broad XRD peak at ~23.7^{o}, attributable to the π-π interaction of PPy chains.[3] Upon carbonization, correspond band is sharper and the maximum peak shifts to ~25.3^{o} implying the partial graphitization of sample at high temperature.[3]

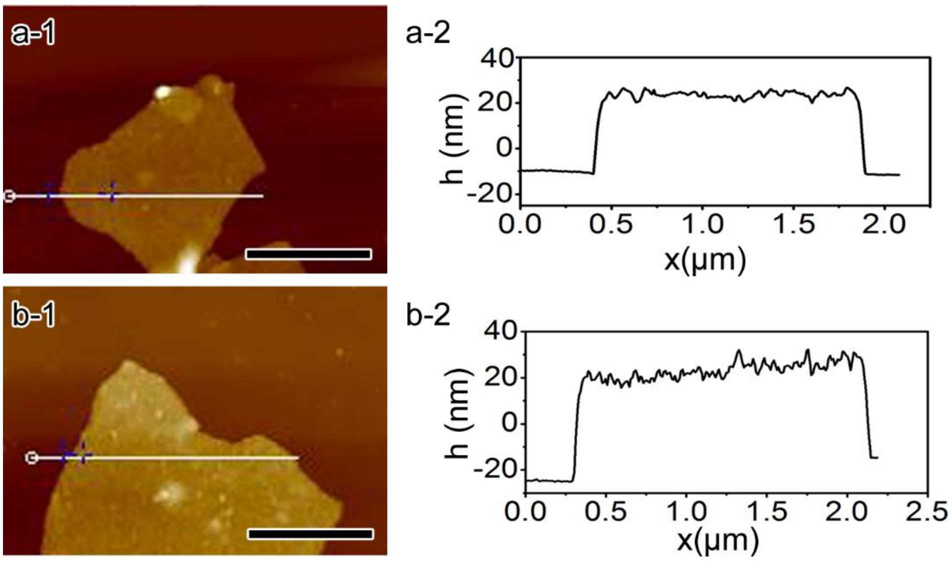

Supplementary Figure 9. AFM survey of mPPy@GO synthesized by BCP templates of PS_{38}-*b*-PEO_{114} (**a**) and PS_{146}-*b*-PEO_{114} (**b**) (scale bar: 1 μm).

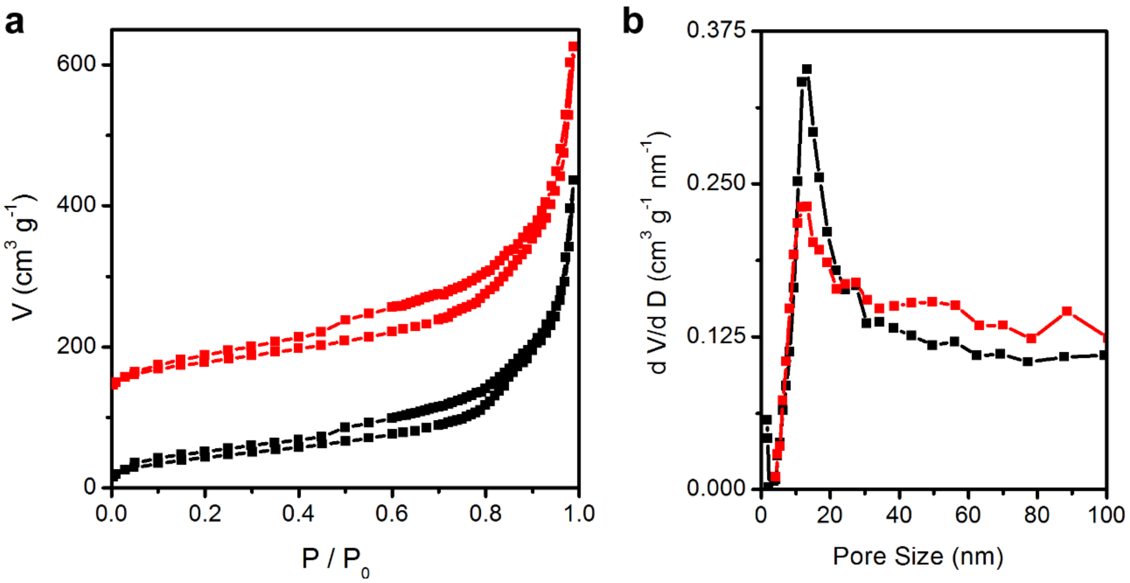

Supplementary Figure 10. Nitrogen adsorption–desorption isotherm (left Figure) and pore size distribution (right Figure) of mesoporous PPy nanosheets synthesized by BCP templates of PS_{102}-*b*-PEO_{114} and subsequently carbonized at 550 °C (black line) and 850 °C (red line) for 2 h in N_2 atmosphere. The isotherm of the sample carbonized at 850 °C is offset vertically by 100 $cm^3\ g^{-1}$ STP.

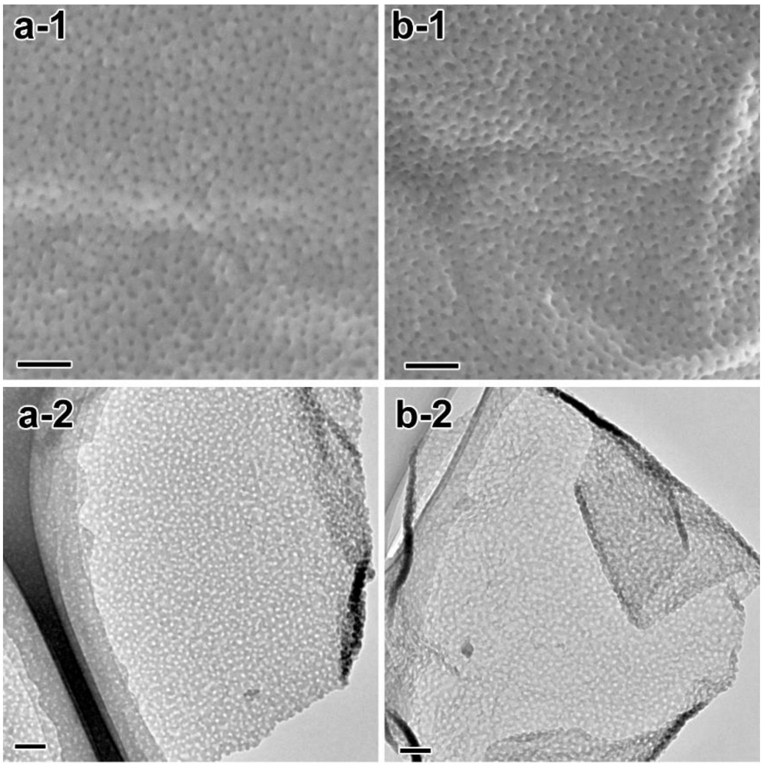

Supplementary Figure 11. SEM (**a-1** and **b-1**) and TEM (**a-2** and **b-2**) images of mPPy@GO synthesized by BCP templates of PS_{102}-*b*-PEO_{114} and carbonized at different temperature in N_2 atmosphere, (**a**) 550 ^oC and (**b**) 850 ^oC (Scale bar: 100 nm).

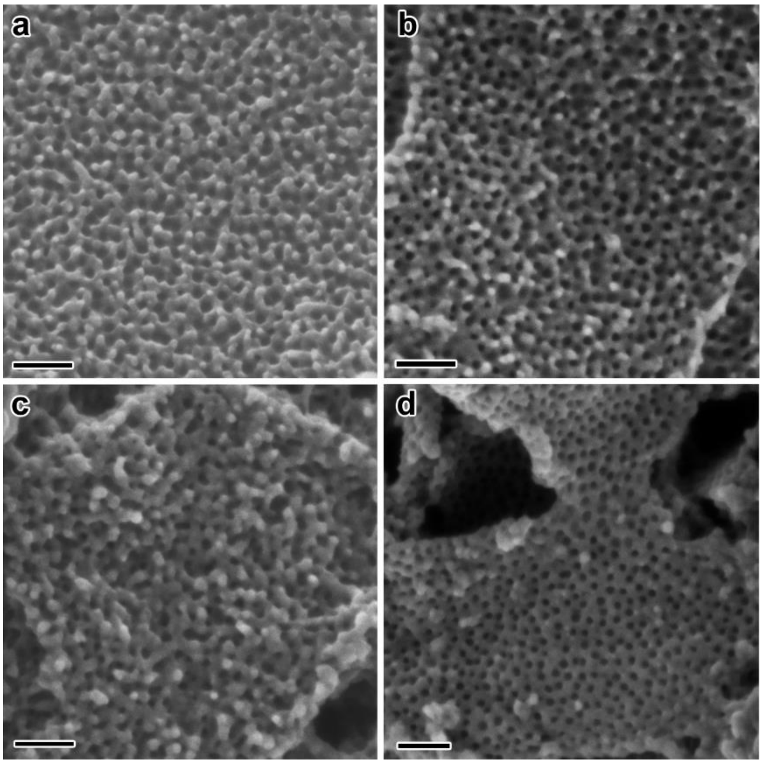

Supplementary Figure 12. SEM images of large-pore mesoporous PANi nanosheets on electrochemically exfoliated graphene (**a**), graphene oxide (**b**), MoS_2 nanosheet (**c**) and titania nanosheet (**d**) (Scale bar: 100 nm).

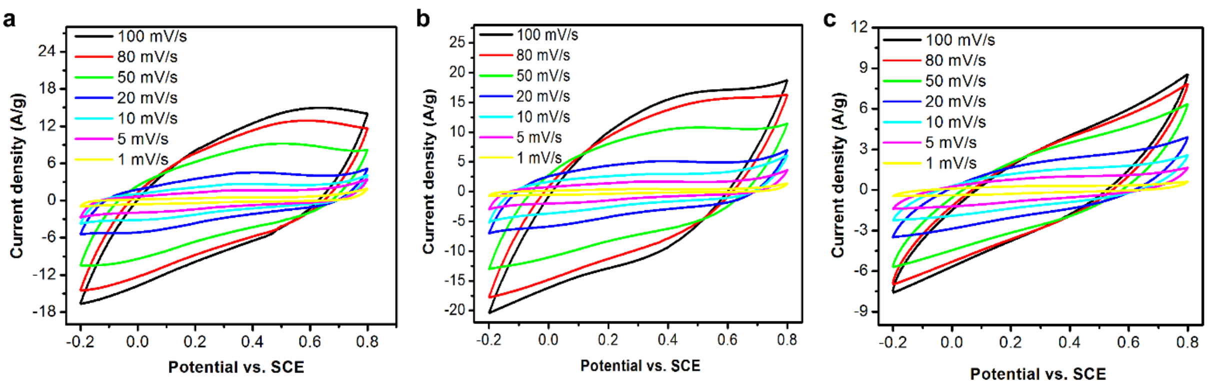

Supplementary Figure 13. CV curves of mesoporous PPy@GO resulting from PS_{102}-*b*-PEO_{114} (**a**), mesoporous PPy@GO resulting from PS_{146}-*b*-PEO_{114} (**b**), and PPy@GO without BCP templates (**c**).

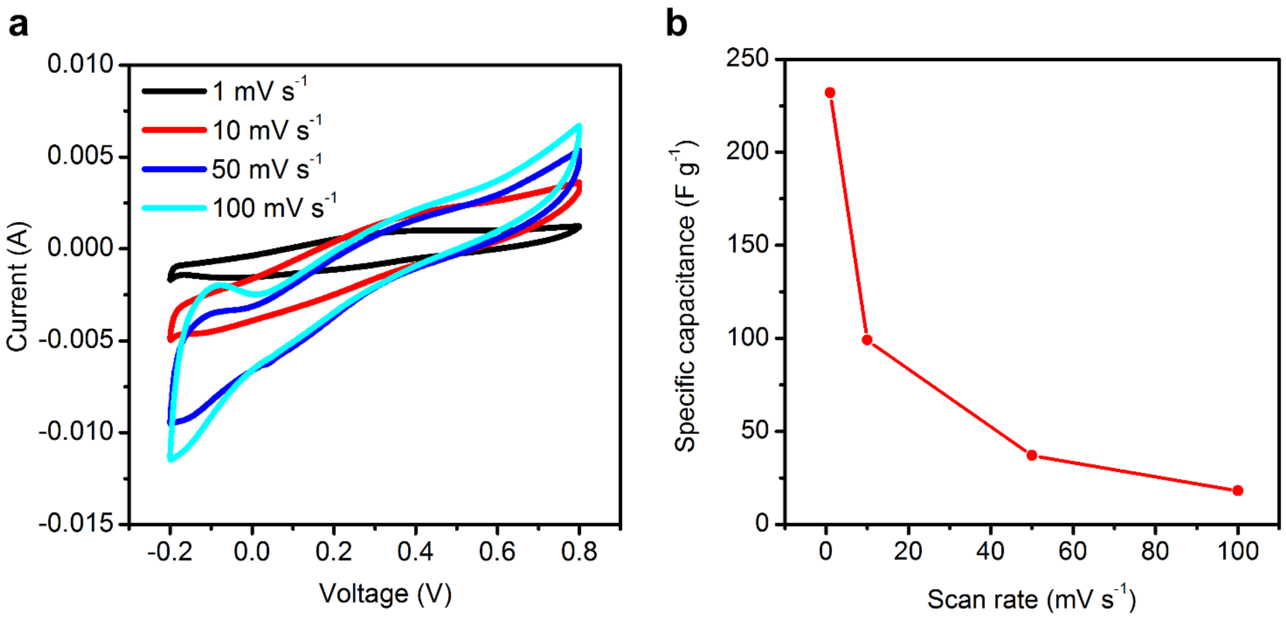

Supplementary Figure 14. Electrochemical performance of 2D large-pore mesoporous mPPy@EG nanosheets. (a) CV curves of mPPy@EG nanosheets synthesized by PS_{102}-*b*-PEO_{114} as electrodes at different scan rates, (b) specific capacitance versus scan rate for mPPy@EG nanosheet electrode materials. Figure R4 shows that mPPy@EG nanosheet electrode materials delivered a slightly lower capacitance and poorer rate performance than those of mPPy@GO under the same conditions. The reason is attributed to that in this approach, 1-pyrenesulfonic acid sodium salt (1-PSA) cannot be completely removed by washing, resulting in the lower capacitance. In addition, the stronger interactions between PPy and GO with respect to PPy and GO would facilitate the structural stability of the mPPy@GO nanosheets and thus render them with better rate performance than that of mPPy@EG.

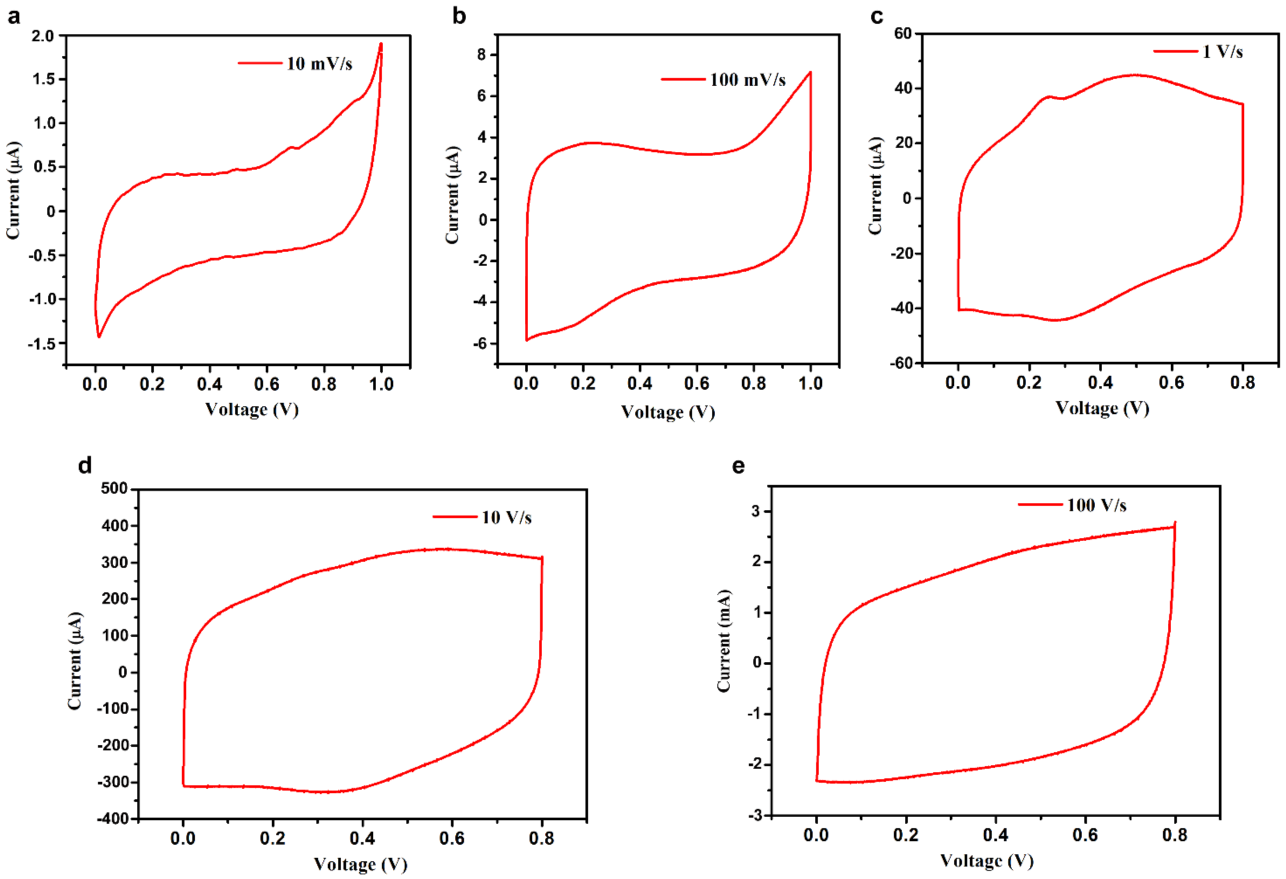

Supplementary Figure 15. CV curves of mPPy@GO-3 nanosheets as electrodes and 1 M H_2SO_4 as electrolyte for micro-supercapacitor at scan rates from 0.01 to 100 V s^{-1}. With 1 M H_2SO_4 as electrolyte, the MSC delivered an areal capacitance of 420 μF cm^{-2} and 60 μF cm^{-2} at scan rate of 10 mV s^{-1} and 100 V s^{-1}, respectively, which is several times higher than the MSC device tested in H_2SO_4/PVA gelled electrolyte. This is because the liquid electrolytes have much higher conductivity than gel electrolytes. Nevertheless, compared with aqueous electrolyte, the polymer gelled electrolyte can reduce the device thickness and weight, as well as simplify the fabrication process because it does not require any special packaging materials, which therefore is commonly used in all-solid-state MSCs.[7-9]

Table 1. XPS data for mPPy@GO.

	C1s			N 1s	
**Position (eV)**	**Area (%)**	**Fwhm (eV)**	**Position (eV)**	**Area (%)**	**Fwhm (eV)**
**284.3**	25.9	1.15	399.8	88.3	1.26
**285.1**	63.7	1.75	401.1	11.7	1.54
**287.2**	6.9	1.7			
**288.8**	3.5	1.3			

Table 2. Porous properties of as-synthesized and carbonized samples of PPy nanosheets with GO.

Samples[a]	Surface area[b] $m^2\ g^{-1}$	Pore volume $cm^3\ g^{-1}$	Pore size[c] nm
mPPy@GO-1	84	0.19	5.8
mPPy@GO-2	85	0.29	13.2
mPPy@GO-3	67	0.23	19.3
mPPy@GO-2-550	165	0.66	13.4
mPPy@GO-2-850	104	0.49	13.4
PPy@GO	24	~[d]	~[d]

Note: [a] Mesoporous PPy nanosheets with GO synthesized by various BCPs of (1) PS_{38}-*b*-PEO_{114} (noted as mPPy@GO-1, the others are similar), (2) PS_{102}-*b*-PEO_{114}, (3) PS_{146}-*b*-PEO_{114}, and carbonized at 550 ^{o}C and 850 ^{o}C in N_2 and contrast samples synthesized without BCP template (PPy@GO). [b] Surface area was obtained based on the Brunauer−Emmett−Teller (BET) method. [c] The pore size was obtained from the nitrogen absorption isotherms based on the BJH method. [d] No pore and pore volume pore has been detected.

Table 3. Overview of chemically synthesized PPy-graphene materials for supercapacitors.

**Electrode materials**	**Specific capacitance(F g^{-1})**	**Reference**
Pure PPy	159	[10]
This work	***385***	-
PPy@Graphene foam	350	[11]
PPy nanoparticles@GO	332	[12]
PPy nanosheets@ Graphene	318	[13]
PPy nanosheets@ GO (electrochemical deposition)	356	[14]
PPynanosheetss@sulfonated Graphene (electrochemical deposition)	285	[15]
PPy nanowires@GO	165	[16]
PPy nanowires@GO	500	[17]
PPy@ hydrazine reduced RGO	387	[10]
PPy@ ethylene glycol reduced RGO	420	[10]

Supplementary References

1 Long, D. *et al.* Preparation of Nitrogen-Doped Graphene Sheets by a Combined Chemical and Hydrothermal Reduction of Graphene Oxide. *Langmuir* **26**, 16096-16102 (2010).

2 Forsyth, M. & Truong, V. T. A study of acid/base treatments of polypyrrole films using 13C n.m.r. spectroscopy. *Polymer* **36**, 725-730 (1995).

3 Liu, S., Duan, Y., Feng, X., Yang, J. & Che, S. Synthesis of Enantiopure Carbonaceous Nanotubes with Optical Activity. *Angew. Chem. Int. Ed.* **52**, 6858-6862 (2013).

4 Atanasoska, L., Naoi, K. & Smyrl, W. H. XPS studies on conducting polymers: polypyrrole films doped with perchlorate and polymeric anions. *Chem. Mater.* **4**, 988-994 (1992).

5 Menon, V. P., Lei, J. & Martin, C. R. Investigation of molecular and supermolecular structure in template-synthesized polypyrrole tubules and fibrils. *Chem. Mater.* **8**, 2382-2390 (1996).

6 Liang, W., Lei, J. & Martin, C. R. Effect of synthesis temperature on the structure, doping level and charge-transport properties of polypyrrole. *Synth. Met.* **52**, 227-239 (1992).

7 El-Kady, M. F., Strong, V., Dubin, S. & Kaner, R. B. Laser Scribing of High-Performance and Flexible Graphene-Based Electrochemical Capacitors. *Science* **335**, 1326-1330 (2012).

8 Wu, Z. S., Parvez, K., Feng, X. & Müllen, K. Graphene-based in-plane micro-supercapacitors with high power and energy densities. *Nat Commun* **4**, 2487 (2013).

9 El-Kady, M. F. & Kaner, R. B. Scalable fabrication of high-power graphene micro-supercapacitors for flexible and on-chip energy storage. *Nat Commun* **4**, 1475 (2013).

10 Liu, Y. *et al.* Ethylene glycol reduced graphene oxide/polypyrrole composite for supercapacitor. *Electrochim. Acta* **88**, 519-525 (2013).

11 Zhao, Y. *et al.* Highly Compression-Tolerant Supercapacitor Based on Polypyrrole-mediated Graphene Foam Electrodes. *Adv. Mater.* **25**, 591-595 (2013).

12 Fan, L.-Q. *et al.* Asymmetric supercapacitor based on graphene oxide/polypyrrole composite and activated carbon electrodes. *Electrochim. Acta* (2014).

13 Xu, C., Sun, J. & Gao, L. Synthesis of novel hierarchical graphene/polypyrrole nanosheet composites and their superior electrochemical performance. *J. Mater. Chem.* **21**, 11253-11258 (2011).

14 Zhu, C., Zhai, J., Wen, D. & Dong, S. Graphene oxide/polypyrrole nanocomposites: one-step electrochemical doping, coating and synergistic effect for energy storage. *J. Mater. Chem.* **22**, 6300-6306 (2012).

15 Liu, A., Li, C., Bai, H. & Shi, G. Electrochemical Deposition of Polypyrrole/Sulfonated Graphene Composite Films. *J. Phys. Chem. C* **114**, 22783-22789 (2010).

16 Biswas, S. & Drzal, L. T. Multilayered Nanoarchitecture of Graphene Nanosheets and Polypyrrole Nanowires for High Performance Supercapacitor Electrodes. *Chem. Mater.* **22**, 5667-5671 (2010).

17 Zhang, L. L., Zhao, S., Tian, X. N. & Zhao, X. S. Layered Graphene Oxide Nanostructures with Sandwiched Conducting Polymers as Supercapacitor Electrodes. *Langmuir* **26**, 17624-17628 (2010).
